# The utility of biomarkers in traumatic brain injury clinical management

**DOI:** 10.1186/s13054-016-1545-5

**Published:** 2016-11-18

**Authors:** Ana Rodríguez-Rodríguez, Juan José Egea-Guerrero

**Affiliations:** 1 Emergency Department, Virgen del Rocío University Hospital, IBIS/CSIC/University of Seville, Seville, Spain; 2 NeuroCritical Care Unit, Virgen del Rocío University Hospital, IBIS/CSIC/University of Seville, Avda. Manuel Siurot s/n, 41013 Seville, Spain

We read the article by Thelin et al. entitled “Utility of neuron-specific enolase in traumatic brain injury; relations to S100B levels, outcome, and extracranial injury severity” [1] with great interest. The authors conclude that while S100B and neuron-specific enolase (NSE) are both biomarkers for long-term outcome, S100B is a more accurate predictor.

These findings concur with the results of a previous study by our research group [2], which showed that serum S100B levels in severe traumatic brain injury (TBI) patients had a higher prognostic capacity to predict mortality than NSE. Similarly, most studies examining the role of diverse biomarkers in TBI pathology have identified S100B as the most promising, with the highest prognostic ability to predict short/long-term mortality [3, 4]. In recent years, researchers have searched for the ideal diagnostic biomarker that could guide TBI treatment in both primary and secondary phases. In TBI, the primary injury is caused by biomechanical damage and the secondary insult is the result of biochemical cascades triggered by damaged neurons, glial cells, and blood vessels. The early rise in biomarkers of secondary injuries could help physicians prevent, or at least reduce, the extent of this potential damage.

Currently, TBI management is guided by clinical histories and neuroimaging techniques. While these techniques may be advanced, they are more costly than serum analysis, involve exposure to ionizing radiations, and have certain limitations when assessing brain damage severity. The ideal biomarker would stratify patients based on their severity, identifying patients with poorer prognosis and greater need for treatment before the patient’s condition worsens. In terms of the nature of the sample, the ideal biomarker could be detected in serum or urine (a waste fluid) in order to avoid cerebrospinal fluid extraction, as reflected in our findings on the role of urine S100B levels as an early predictor of mortality after severe TBI [5]. Yet, the variability of potential inclusion criteria in clinical analysis, and particularly in clinical trials, hinders the standardization of biomarker utility. Studies with such promising results, like that of Thelin et al. [1], encourage researchers to continue investigating TBI pathophysiology, to find the perfect biomarker for TBI assessment, or at least a set of biomarkers that together can reflect the diverse injury characteristics of TBI.

## Abbreviations

NSE: Neuron-specific enolase
TBI: Traumatic brain injury
