# Supplementary material for: The utility of biomarkers in traumatic brain injury clinical management
Source: Crit Care. 2016 Nov 18;20:376. doi: 10.1186/s13054-016-1545-5 (PMC5114747; doi:10.1186/s13054-016-1545-5)
Supplement: Related texts

# Reply: Authors’ response

**Authors:** Eric Peter Thelin, Emma Jeppsson, Bo-Michael Bellander, David W. Nelson

Dear Drs. Rodríguez-Rodríguez and Egea-Guerrero

We appreciate that you took the time to read our recently published article in *Critical Care* [1].

We agree with the authors concerning the findings in their recent publication “S100B and neuron-specific enolase as mortality predictors in patients with severe traumatic brain injury” [2], which unfortunately was not available to us when we prepared our manuscript. In addition to mortality, we looked at the different stages of the Glasgow Outcome Score and noted similar results where higher serum levels of both S100B and NSE correspond to a more unfavourable outcome.

However, we would also like to stress that further monitoring (after 72 h) of unconscious TBI patients is of value, which we showed in a cohort of n = 250 patients in whom secondary increases of S100B were correlated to newly developed radiological findings and subsequently a worse outcome [6]. Moreover, these secondary increases have been shown to aid in clinical decision making for these patients by other groups [7]. Drs. Rodríguez-Rodríguez and Egea-Guerrero mention urinary S100B as a potential marker of injury assessment, and while we appreciate additional compartments could be sampled, we would like to stress the need for further research in order to create valid thresholds and temporal patterns of release for these.

In unison with Drs. Rodríguez-Rodríguez and Egea-Guerrero, we stress that the field should continue to utilize biomarkers to better understand how to interpret and assess levels in bodily fluids and how they relate to injury severity and outcome. We suggest that future promising markers of brain tissue fate be compared with S100B, as it appears to have the most utility of the TBI markers studied to date, in order to determine how they can add additional information concerning outcome and assessment of injury.
